# Business–nonprofit hybrid organizing: a dynamic approach to balancing benefits and costs

**DOI:** 10.3389/frhs.2023.1164072

**Published:** 2023-05-23

**Authors:** Emma-Louise Aveling, Jane E. Roberts, Lauren A. Taylor, Nazmim Bhuiya, Sara J. Singer

**Affiliations:** ^1^ Department of Health Policy and Management, Harvard T. H. Chan School of Public Health, Boston, MA, United States; ^2^ Department of Population Health, New York University School of Medicine, New York, NY, United States; ^3^ Massachusetts Department of Public Health, Boston, MA, United States; ^4^ Department of Medicine, Stanford University School of Medicine and Graduate School of Business, Stanford, CA, United States

**Keywords:** collaborative forms, hybrid organizing, cross-sector collaboration, qualitative, business

## Abstract

**Introduction:**

Efforts to address complex public health challenges can benefit from cross-sector collaboration, while also fostering growing business sector engagement in promoting health equity. What form business-nonprofit collaboration should take, however, is a difficult question for managers and leaders. Hybrid organizational forms, which combine for-profit and nonprofit elements within a single organization in unconventional ways, offer an innovative and potentially promising approach. Yet, while existing typologies of cross-sector collaboration have identified hybrid forms at one end of a continuum of possible forms of collaboration, these typologies do not differentiate the diversity such hybrid forms may take, and the costs and benefits of these innovative hybrid forms are poorly understood. This leaves managers interested in promoting public health through business-nonprofit hybrid organizing with limited guidance about how to maximize potential merits while mitigating drawbacks.

**Methods:**

We performed a qualitative comparative case study of three examples of business-nonprofit hybrid organizing. Data collection included 113 interviews with representatives from 42 organizations and observation of case study activities. We used thematic analysis within and across cases to characterize the form of hybrid organizing in each case and to examine benefits and costs of different forms for supporting initiatives.

**Results:**

We identified two hybrid, collaborative forms - Appended and Blended forms. Each form had benefits and costs, the significance of which shifted over time contingent on changing strategic priorities and operating environments. Benefits and costs of particular forms become more or less important for establishing and sustaining initiatives under different conditions, requiring a dynamic view.

**Discussion:**

No particular form of business-nonprofit hybrid organizing is inherently better than another. Optimizing hybrid organizing and ensuring resilient collaborations may mean allowing collaborative forms to evolve. Practitioners can manage tradeoffs between benefits and costs through an ongoing process of assessing the fit between a given collaborative form, strategic priorities, and relevant features of the operating environment. This dynamic view offers important insights for ensuring the resilience of business-nonprofit collaborative efforts to enhance public health.

## 1. Introduction

The pandemic has underscored the significant potential for business to contribute to cross-sector initiatives addressing interrelated social, economic, and environmental drivers of health (1). Complex public health challenges, such as health inequities or food insecurity, cannot be addressed by any single sector acting alone (2). Innovative, cross-sector initiatives involving business and nonprofits represent an important opportunity for the private sector to contribute to public health. Such cross-sector initiatives could benefit from the diverse skills, resources, and knowledge for-profit businesses may bring, while also offering valuable opportunities to foster the growing appetite for business sector engagement in promoting health equity (3). However, what specific form this collaboration should take is a difficult question for private sector managers and leaders to answer. “Hybrid” organizational forms, which combine for-profit and nonprofit elements within a single organization in unconventional ways, offer an innovative and potentially promising approach to harness cross-sector collaboration for public health (4). Yet to date, insufficient attention has been paid to the diversity of forms such deeply integrated, hybrid collaborations may take (4, 5). Moreover, the relative benefits and costs of different hybrid forms are poorly understood. This leaves business managers interested in hybrid forms of business–nonprofit collaboration with limited guidance regarding how to maximize the potential merits for establishing and sustaining initiatives, while mitigating the drawbacks. In this paper, we draw on qualitative case studies of business–nonprofit hybrid organizing to identify lessons for optimizing this approach to supporting what we call “social purpose initiatives,” i.e., initiatives targeting social, economic, and environmental factors that constitute critical foundations of public health.

## 2. Background

Collaboration between business and nonprofit organizations is well-established in practice and scholarship (6–8) and is a prominent feature of efforts to advance equitable public health on a global scale (2, 9). Such collaborations offer the potential to pool complementary resources to achieve more than either could alone by drawing on the strengths and mitigating the weaknesses of each sector (6). Changing demands and expectations for both sectors are fostering increasingly integrated forms of collaboration (10, 11). Drivers include increasing “institutional complexity,” i.e., incompatible prescriptions for organizational norms and practices (12, 13); growing demands for business to demonstrate social responsibility to various stakeholders, including contributions to the health and wellbeing of societies in which they operate, and to do so in authentic and holistic ways that go beyond just philanthropy^1^; and intensified competition for resources among nonprofits (14). At their most integrated form, business–nonprofit collaborations may involve novel forms of hybrid organizing that blur sectoral boundaries and diverge from traditional business or nonprofit models (10). While hybrid business–nonprofit organizing offers promising innovation, it remains unclear which form may best support and sustain social purpose initiatives in a changing environment, so that they can make the desired contributions to public health.

### 2.1. From business–nonprofit interaction to hybrid organizing

Business–nonprofit collaboration is typically conceptualized on a continuum, ranging from time-limited philanthropy to deep, transformational integration of business and nonprofit resources, activities, norms, and managerial and governance structures (6). The integrated end of this spectrum can result in hybrid organizing, i.e., forms of business–nonprofit integration that combine for-profit and nonprofit organizational elements within a single organization in unconventional ways while maintaining a mixture of market- and mission-oriented practices, identities, norms, and rationales (15).

The literature on cross-sector collaboration says little about the diversity of forms that this type of deeply integrated, hybrid collaboration might take (5), although it *can* take many forms. Hybrid organizations may be legally structured as for-profit, nonprofit, or both. They can vary in the amount and configuration of integration of business and nonprofit elements, for example, in the degree of compartmentalization vs. merging of elements such as structures, practices, people, or identities (16–18). Some organizational scholars argue that hybrid forms are so dynamic that hybrid *organizing*—the verb—is a more accurate conceptualization than hybrid organizations (4). In addition to the lack of elaboration of the different forms hybrid organizing can take, the relative costs and benefits of these different forms have not yet been well understood (19).

### 2.2. Business–nonprofit hybrid organizing: a double-edged sword

Establishing and sustaining social purpose initiatives requires securing resources (e.g., funding, physical assets) and building relationships with key stakeholders (e.g., operational partners, funders). Existing literature suggests that hybrid organizing can create benefits and costs related to both these organizational needs, making the value of hybrid collaborative forms double-edged. If costs outweigh benefits, and/or collaborative efforts cannot be sustained, organizations cannot fulfill their mission, and synergistic benefits of collaboration for public health will not be achieved (2).

Hybrid organizing's ability to combine elements that “would not conventionally go together” (4) is the source of both its potential benefits and challenges (10). Hybrid organizing has the potential to achieve a “best-of-both-worlds” win for public health, enabling access to expanded resources and synergistic benefits of combining knowledge, skills, and expertise from nonprofit and business sectors (20). Simultaneously, differences in the assumptions, values, and norms of each sector—in institutional “logics”—can generate conflict, misunderstandings, and hinder organizational functioning and therefore the ability to accomplish an organization's health and social mission (12). Resource dependency theory (21) highlights the potential with hybrid organizing for imbalances in power and resources to result in the dominance of one element at the expense of another, e.g., dominance of business priorities over mission (22), attenuating social and public health benefits.

Central to securing both resources and productive relationships is the extent to which an organization is perceived as legitimate (23), i.e., its actions are perceived to be “desirable, proper, or appropriate within some socially constructed system of norms, values, beliefs, and definitions” (24). Hybrid organizing has been described as both a risk to and a strategy for securing and sustaining organizational legitimacy. On the one hand, business–nonprofit hybrid organizing may enable organizations to satisfy the expectations of a broader spectrum of stakeholders, thus helping to secure legitimacy to pursue organizational missions that include public health goals (12). On the other, where the combination of business and nonprofit elements violate established expectations for what is considered socially sanctioned organizational behavior, such as when a nonprofit appears to act too much like a business, initiatives may experience a legitimacy discount in the eyes of important stakeholders (25)—undermining its potential to secure resources and relationships needed to accomplish its public health goals.

Most prior research has focused on the tensions stemming from conflicting underlying logics that threaten the sustainability of hybrid organizing (19), and many factors affecting capacity to manage such tensions internally have been identified, including the following: the importance of strong, trusting interpersonal relationships; degree of alignment in partners' goals and interests (6, 8); value of boundary spanners and ambidextrous leaders able to bridge business and nonprofit worlds (12, 26); and microlevel processes supporting effective communication and safe spaces for negotiation (4, 27). These insights have been derived from and applied to diverse forms of hybrid organizing. Yet, the degree to which these costs and benefits vary across different forms of business–nonprofit hybrid organizing, what their implications are for establishing and sustaining initiatives targeting socioeconomic drivers of health, and how costs/benefits may vary given the contextual specifics of initiatives are less clear (5, 19). This is particularly problematic given the inherently double-edged nature of hybrid organizing. As such, organization leaders lack guidance about how to optimize business–nonprofit hybrid organizing to support resilient social purpose initiatives.

To address this gap, we conducted in-depth qualitative case studies of innovative business–nonprofit hybrid organizing that supported longstanding social purpose initiatives. These initiatives targeted different public health issues: two initiatives promoted physical activity among public school students to improve physical and socioemotional health and support academic success; the third targeted the lack of access to affordable, healthy food in low-income urban neighborhoods. From these cases, we characterized two distinct forms of business–nonprofit hybrid organizing: an *Appended form*, where a nonprofit unit is embedded within an established business, and a *Blended form*, where a newly established organization seeks to blend nonprofit and business elements throughout all units and activities. We then compared their strengths and weaknesses for supporting social purpose initiatives. Recognizing the potentially double-edged nature of business–nonprofit hybrid organizing, we also examined contextual factors that influenced the relative importance of these costs and benefits over time. From these findings, we outline a dynamic model of how practitioners can balance the trade-offs and optimize hybrid collaborative forms through an ongoing process of assessing fit between the characteristics of a given form, strategic priorities of the initiative, and relevant features of the operating environment. This dynamic view offers important insights for ensuring the resilience of business–nonprofit collaborative efforts and optimizing their value for public health.

## 3. Methods

We conducted three qualitative case studies of business–nonprofit hybrid organizing. Case studies facilitate a holistic perspective on the complex organizational processes within each case (28). By combining 113 interviews with representatives from 42 organizations, including practitioners in different roles in case study organizations, their collaborators, and local leaders from multiple sectors, and observations of case study activities, we triangulated diverse perspectives to gain a rich, in-depth understanding of the dynamics of each case and the contexts in which they operated. A comparative analysis across cases enabled us to move beyond descriptive accounts to identify analytically generalizable insights about the benefits and costs of hybrid organizing that were context-specific (29). We purposefully selected cases with some consistent features: all three cases were based in and served the same city; all three had sustained their social purpose initiative for 6–10 years at the time of research, allowing us to learn from successful cases and to take account of the dynamic nature of cross-sector, hybrid organizing over time (7). Cases were diverse in terms of business sector and the health focus of the initiative; we also purposefully selected cases that appeared to combine business and nonprofit elements in different ways—although characterizing the hybrid form was an aim of the investigation. Data collection took place between February 2018 and December 2019.

The research context was a medium-sized coastal city in the United States. The city and metropolitan area host many regional, national, and international companies in diverse sectors (from finance to clothing), including large health, technology, and education sectors. While the region enjoys relatively high levels of economic mobility and health, the city itself suffers significant inequity across racial and neighborhood lines.

The study received ethical approval from the [Institution suppressed] Institutional Review Board. Senior organizational leaders agreed to the participation of organizations in the study, and interview participants provided individual informed consent. To protect participants' identities, we provide limited details on the organizations and location involved.

### 3.1. Data generation

For each case study, we interviewed members of the focal organization and its collaborating organizations and conducted observations. Details of these 75 interviewees and 16.5 h of observations are provided in Table 1. Within each case, we identified potential interview participants and observation opportunities in consultation with senior leaders from the focal organization. We interviewed 22, 32, and 21 individuals from cases one, two, and three, respectively. Interviewees included board members, core business staff (e.g., marketing staff), and staff primarily involved in the social purpose initiative (e.g., directors of development), from senior management (e.g., CEOs) to frontline roles (e.g., shopfloor, fieldworkers). Collaborating stakeholders included operational partners (e.g., staff of public schools where the initiative was being run, suppliers to the retail nonprofit), sponsors, and funders. While we cannot know whether case study gatekeepers steered us away from particular members or stakeholders, our data did include critical perspectives. Observations (of initiative activities, workspaces, and stakeholder meetings) provided alternative perspectives on the nature of hybrid organizing and organizational settings (e.g., physical spaces; interpersonal dynamics).

**Table 1:** Case study data: participants interviewed, and hours of observation, for each of three case studies.

Data generation method	Data source		*N*=
Case study 1: Appended form			
Observations	Initiative activities, office-based activities, staff meetings		**6.5 h**
Semistructured interviews	*Hybrid organization members*	Core business—senior and middle managers	6
		Social purpose initiative—senior and middle managers, frontline staff, board members	12
	*External collaborators (operational partners)*	Public sector organizations—senior managers, school-based staff	4
Total number of interview participants			**22**
Case study 2: Appended form			
Observations	Initiative activities, office-based activities, staff meetings		**5 h**
Semistructured interviews	*Hybrid organization members*	Core business—Senior managers, staff, board members	6
		Social purpose initiative—Senior and middle managers, staff	9
	*External collaborators (Funders, operational partners)*	Public sector organizations—senior managers, school-based staff	9
		Nonprofit organizations—Senior leader, manager, board member	3
		For profit organizations—Senior managers, staff	5
Total number of interview participants			**32**
Case study 3: Blended form			
Observations	Shop floor, on-side educational activities, community meeting		**5 h**
Semistructured interviews, focus group	*Hybrid organization members*	Senior and middle managers	7
		Board members	3
		Store managers	3
		Frontline staff (1 × focus group)	4
Semistructured interviews	*External collaborators (funders, operational partners)*	Nonprofit organizations—senior managers of local foundation, health, and social service organizations	4
Total number of interview and focus group participants			**21**
Total interview participants			**75**
Total hours of observation			**16.5**

Bolded values correspond to the text in the cell directly to the left.

We also interviewed, as part of a larger study of cross-sector collaboration within the city, 38 local leaders from business, nonprofit, and public sectors with the experience of cross-sector collaboration (Table 2). For this paper, we used these interviews to deepen understanding of the operating environment of our case studies.

**Table 2:** Local context interviews: interviews with local sector stakeholders involved in (noncase study) cross-sector organizing.

Organization type	Individual roles	Number
For profit: financial services and media sectors	Senior and middle managers (CSR, Marketing depts.)	11
Nonprofit: local organizations with diverse missions, from art, sport to youth empowerment; one university and one local business association	Senior managers, frontline staff, managers	25
Public Sector	One elected and one appointed city official	2
Total interview participants		38

We conducted interviews using a semistructured guide, adapted to reflect interviewees' diverse roles. Questions covered organizational and social purpose initiative missions; roles, strengths, and limitations of different organizational units, stakeholders, and collaborating organizations and their contributions to the initiative; factors influencing the dynamics of business–nonprofit hybrid organizing, including national and local contexts. Interviews, conducted in person (in private workspaces) or by phone, lasted 33–98 min (average 56 min) and were audio-recorded and transcribed verbatim.

### 3.2. Analysis

We analyzed data using reflexive thematic analysis (30), which involved generating themes through iteratively synthesizing systematic, open coding with existing concepts from relevant theoretical and empirical literature on business–nonprofit collaboration, hybrid organizing, and institutional theory. Our analysis (supported by NVivo software) was oriented to (1) characterizing the form of hybrid organizing in each case, and (2) examining the context-specific benefits and costs of these different forms for establishing and sustaining initiatives.

To characterize the form of hybrid organizing in each case, we first analyzed data within-case*,* to create a descriptive account of how nonprofit and business elements were combined (e.g., in terms of governance, resource flows, interactions between units and staff). Subsequent cross-case interpretive, comparative analysis was oriented to characterizing common and contrasting features of the different forms of hybrid organizing, resulting in distinguishing two forms (Appended and Blended) across the three cases. This interpretive work was informed by existing typologies (17–19, 31).

To understand the benefits and costs of each form, we first coded data descriptively within case. We also coded the entire data set descriptively to capture and characterize the environment. We triangulated these descriptive accounts to compare and contrast costs and benefits and to identify cross-cutting themes. Through iterative cross-case comparison, informed by existing literature on hybrid organizing and institutional complexity (4, 12), we grouped patterns of benefits and costs around two main themes: material and relational resources. Analyses are presented using illustrative quotes, anonymized to protect individual and organizational identities.

## 4. Findings

We identified two distinct forms of business–nonprofit hybrid organizing—an *Appended form* (encompassing two case studies) and a *Blended form*—which we describe in Section 1. In Section 2, we describe the benefits and costs associated with each form and how these changed over time contingent on context, underscoring the need for a dynamic approach to sustain social purpose initiatives and pursue public health goals.

### 4.1. Two forms of business–nonprofit hybrid organizing

We differentiated Appended and Blended forms of hybrid organizing.

#### 4.1.1. The Appended form

The Appended form of hybrid organizing entailed the coexistence of a nonprofit unit *within* a business. Each of our two cases of the Appended form (Case 1 and Case 2) combined operational and managerial integration of business and nonprofit elements with a degree of differentiation. Thus, distinct business- and nonprofit-conforming organizational elements were maintained, while the social purpose initiative was supported through resources and assets derived from both.

The initiatives in both Appended cases had been operational for approximately 10 years at the time of study and were both delivered in public schools. Both aimed to improve students' physical, socioemotional health, and school success through opportunities for physical activity and other forms of support. Organizations hoped that this would help reduce “gaps in academic achievement,” with disparities in educational outcomes seen as one local driver of inequities in public health. Case 1 was established by the CEO of a large, privately owned industrials company, in response to the needs identified within the local public school district. Over 10 years, the initiative was formalized as a nonprofit housed within the business, and then, in the last 2–3 years, spun out to become increasingly independent and eventually registered as a 501c3 (charitable nonprofit). Case 2 started out as a small, community-led initiative. After approaching an international retail corporation to seek sponsorship, its founders and the corporation CEO agreed to bring the initiative in-house as the business's signature social purpose initiative. One of the founders became director of all the business's social purpose activities, with this initiative accounting for ∼90% of the business's social purpose funding. The initiative was housed within the business's foundation, which was colocated with the business's headquarters.

Business–nonprofit integration was reflected in colocation; significant staff interaction at multiple levels and shared managerial arrangements (e.g., social purpose initiative staff reporting to senior managers of the business, business staff occupying seats on the initiatives' boards); involvement of business employees in a broad range of activities supporting the initiative (e.g., weekly meetings, fundraising, IT support, volunteering). The for-profit “brand” and the social purpose initiative in each organization remained differentiated legally and structurally: the initiatives operated as discrete units with their own staff, workflows, management hierarchy, and advisory boards. Moreover, as described below, staff associated with business and nonprofit elements each sought to operate in accordance with the distinct norms and logics of their respective sectors.

Members described this approach as an opportunity to “get the best of both worlds” by conforming to normative expectations for both sectors, i.e., achieving the initiatives' mission would be best supported if the business elements did good business and the nonprofit staff carried out its roles in accordance with nonprofit best practices. Although not always easy to realize in practice (as we will describe), commitment to this Appended approach was epitomized in reports that staff embodying each element regarded each other as “experts in [their] own space” (Case 2, manager), while senior leaders from both business and nonprofit sides had a place at the table in determining the strategic direction of initiatives.

#### 4.1.2. The blended form

The Blended case (Case 3) was a grocery retail nonprofit whose mission was to improve access to healthy, affordable food in low-income communities. Rather than compartmentalizing and maintaining a distinction between business and nonprofit organizational elements, the Blended form sought to integrate nonprofit and business norms and practices across all units and structures of the organization into a novel, “unified and consistent framework for cognition and action” (4).

The grocery retailer was incorporated as a 501c3 (charitable) nonprofit that received philanthropic grant funding and also generated revenue through the sale of goods. Its CEO, who had a business background, recruited several senior managers with business degrees and a grant manager with experience of the nonprofit sector. Reduced cost or donated stock was acquired from food wholesalers, in line with dietary guidelines (excluding foods exceeding certain sugar, sodium, or fat content thresholds), and sold in the Blended organization's stores at substantially below-market prices.

The senior management's goal was to eventually break-even, with retail activities generating sufficient funding to sustain the organization, ending reliance on philanthropic grants and donations. Its founders believed that this was the unique value proposition that hybrid organizing offered—namely, that it was an organization that could reduce local food insecurity and be self-sustaining through its own sales. As one board member put it, breaking even was what made this organizational model “more intriguing” than simply “providing low-income people with food.” In this sense, the Blended form sought to realize the transformative potential of diverging from both nonprofit and business norms, not only to get the best of both worlds but also to overcome the relevant weaknesses of each sector. On the nonprofit side, this meant reducing precarious dependence on winning grants; on the business side, it was avoiding traditional food retail market issues, such as lack of access to healthy food in low-income neighborhoods. “We've got to find collaborative solutions to a systemic problem. We are not a typical grocery store. […] One of the ways we're a force for good is we don't carry soda, cookies, cakes, pies. […] even if they would be high margin and add to sales, we sacrifice that” (Case-3, 018, Manager).

In practice, seamlessly blending business and nonprofit logics throughout the structures, systems, and practices of the organization proved challenging, as we will describe. Operational for 6 years at the time of research, the organization had yet to reach its goal of breaking even.

### 4.2. Contingent benefits and costs associated with different forms of hybrid organizing

**Figure 1:**
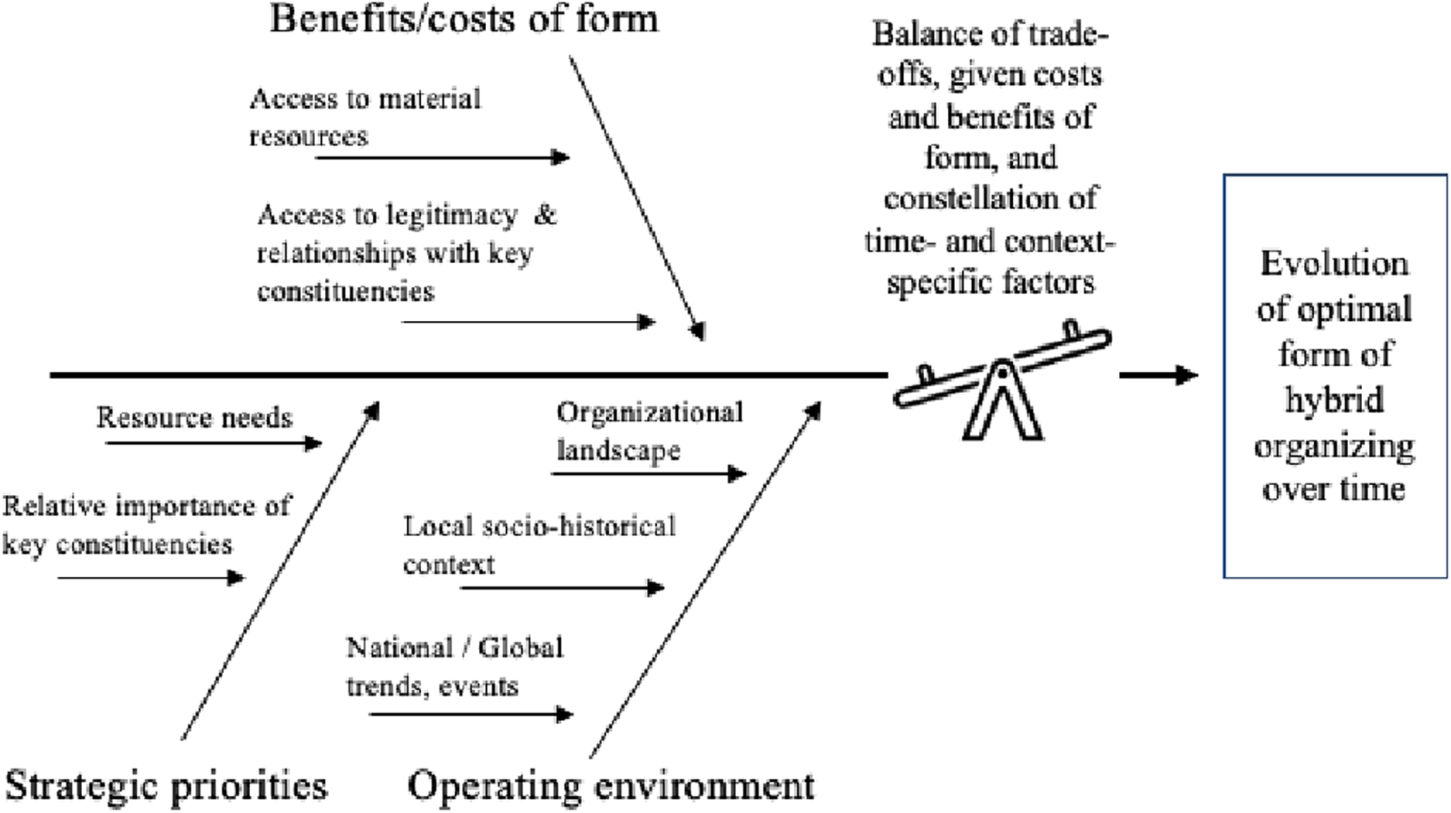
A dynamic model of optimizing hybrid organizing: maintaining fit between the costs and benefits of the collaborative form, and contextual and temporal contingencies.

Appended and Blended forms experienced benefits and costs associated with business–nonprofit hybrid organizing, as summarized in Table 3. The significance of these costs and benefits for securing the necessary resources, relationships, and legitimacy shifted over time contingent on the changing strategic priorities of each initiative and aspects of the environment in which they were operating (Figure 1). Organizational configurations of business–nonprofit integration were thus not static. Rather, case trajectories reflected recognition of the need to evolve in order to achieve their missions, with participants weighing up the potential pros and cons of both, more and less integration of business and nonprofit elements.

**Table 3:** Costs and benefits of Appended and Blended forms of business–nonprofit hybrid organizing.

	Appended form	Blended form
Benefits	*Material:* Reliable, large-scale financial support from (internal) business activities; expanded pool of potential resources through leveraging existing business networks (including clients, employees, contractors, and executives’ personal networks); in-kind support (e.g., business employee volunteer time, “back-office” support).	*Material:* Potential to rely on a secure source of internally generated revenue from retail activities, reducing reliance on competing for grants; expanded pool of externally sourced support through leveraging organization members’ cross-sectoral networks (e.g., board members’ networks in the business community).
	*Relational:* Reputational halo of association with credible business helps broker implementing relationships locally; potential to win legitimacy through a distinct social purpose unit that conforms to a recognizable nonprofit form.	*Relational:* Appeal to some external audiences (e.g., business community, social entrepreneurs, nonprofit partners) on the basis of an atypical, innovative form, committed to social good but with the potential to be self-sustaining.
Costs	*Material:* Close association with wealthy business may deter other funders; over-reliance on internal funding can stymies fundraising capacity; difficulties prioritizing social purpose over business needs (e.g., for back-office support).	*Material:* Startup nature means revenue limited at start and covering costs relies on the successful growth of revenue/retail business.
	*Relational:* Being embedded within established business can compromise the ability to appear to conform to the norms of the nonprofit sector (e.g., due to the dominance of language, practices, and processes characteristic of the business sector).	*Relational:* Non-conformity to *either* a “pure” business or a nonprofit form (neither regular grocers nor recognizable food charities) limits appeal to some external audiences, e.g., target consumers.

#### 4.2.1. Contingent benefits and costs of the Appended form

For Case 1 and Case 2, a key strategic priority in the early years was to gain visibility in the local organizational landscape and secure relationships with operational partners (local public schools) so that their business–nonprofits could establish the initiatives and demonstrate positive impacts. Over time, their priorities shifted to scaling-up and expanding into schools in other regions.

The local, organizational context was characterized by a crowded nonprofit landscape, with many well-known and long-established nonprofits, and a small, “tight knit,” “philanthropically inclined” business community with dense ties across business, nonprofit, and public sector networks. In this context, the strengths of the Appended form were particularly valuable in the initiative's nascent phase. Initially, given the limited, local scale of the initiatives, *material* resources derived from the large, well-established businesses provided secure, reliable funding sufficient to cover the majority of the initiatives' costs. This was particularly valuable as initiatives did not yet have evidence of success to “hang their hat on” (Case 1, Senior manager) and so compete with other nonprofits for funding. In addition, initiatives benefited from businesses' back office operational and infrastructure support (e.g., from business' existing human resources, IT, or legal departments) and from expanded pools of potential donors via the businesses' well-established networks of employees, clients, contractors, and local civic leaders.

The *relational* benefits of Appended hybrid organizing were also high in the nascent phase. The local landscape was seen as challenging for new initiatives to break into and capture the attention of funders and potential board members. Tapping into business' existing networks and associating with a well-respected business and its leadership boosted the initiatives' credibility and profile with local business and civic leaders. At the same time, the hybrid form—being “so unique and different”—was thought to enhance confidence in the businesses as “truly committed to the community” (Case 1, Manager). Further, business leaders could leverage their civic and political connections within the city to broker essential relationships with operational partners in the public school system.

I don't think, if a nonprofit wanted to jump into a dozen schools who was completely unknown without any connection to the city, would a Mayor or the Superintendent say, “Sure, come on in.” So like the relationship and the trust that [the Mayor] and the Superintendent had with [Business CEO] I think allowed for mobility and access. (Case-1, 018, Manager)

However, as the strategic priority for initiatives shifted to scaling up, the value of these Appended form benefits waned. Expansion beyond the original locale where businesses were well networked exceeded what a single business could financially support. As the importance of attracting external funders increased, some of the costs associated with the Appended form became more prominent.

Joining [the business] accelerated [the initiatives]'s growth by about five years. […]. The problem is that that lasted for about the first five years, and now I think being part of [the business] is actually hindering their growth. (Case-2, 028, Manager)

This could be a national program […]. For that aspiration to happen the irony is we [the business] really have to sort of let it go. (Case-1, 025, Manager)

Participants reported that the initiatives’ close association with a wealthy business or CEO created an impression that the initiative did not need external funders, or for other reasons (e.g., competitive business relationships) made it a less attractive funding recipient. Moreover, extensive early reliance on internal funding had stymied development of their capacity for effective external fundraising.

It's a private foundation closely associated with a major brand. Why on earth would anyone else want to give money to that, right? (Case-2, 028, Board member)

The [business] has always served as a backstop from a cash flow perspective, so the program is ahead of the development arm, I think, because of that, so we have a couple year lag. (Case-1, 014, Manager)

At this stage, staff feared that language, practices, or expenditures aligned with business but not nonprofit norms limited their ability to effectively compete for philanthropic funding. Nonprofit staff sought to capitalize on the differentiated structure of the Appended form to offset such legitimacy discounts by conforming to nonprofit norms and standard practices in areas that did not affect core business operations.

*Participant*: It's like literally trying to right size the organization to use nonprofit standards and language. *Interviewer*: Why does it need to be done the way nonprofits do it? *Participant:* I mean, one, it's good because it actually keeps us in line with what high performing nonprofits are doing. […] it also lets people see that we're a thought leader as well in this universe. Our [other] funders wanna see it. (Case-1, 003, Manager)

They [business-side staff] were eventually receptive to us saying, “ […] our funding needs to go to fund schools, not to pay an advertising agency”. (Case-2, 020, Staff)

Nonetheless, asymmetries between business and nonprofit elements meant that business priorities did sometimes prevail, e.g., marketing staff prioritizing work on “the brand” at the cost of the initiative. This particularly hindered the capacity to expand initiatives in line with achieving social purpose goals (e.g., due to organization-wide hiring freezes).

I think some of the drawbacks are that we are confined to this [Business] system from a say hiring perspective, from a growth perspective […] If they go on a hiring freeze, we go on a hiring freeze. (Case-2, 022, Manager)

In both cases, some participants questioned whether continuing in this form was the optimal course for sustaining the initiative and maximizing its impact. In Case 2, most participants acknowledged a sense of dilemma, but opinions were mixed about whether the move should be toward greater or lesser integration:

I think there are two paths: one, [business] starts investing more fully in [initiative] […] the other successful collaboration would be [business] commits to level funding for a five year period, spins [the initiative] out into a standalone public charity. (Case-2, 028, Board member)

In Case 1, although managers had agreed to spin out the initiative into an independent nonprofit organization, given the many perceived advantages of hybrid organizing, efforts toward separation were tentative, and much remained unclear about what form the business–nonprofit relationship would ultimately take.

To play in the middle is pretty hard. […] we hit a crossroads […] that we have been in over the last two to three years of should we just–it's like you really almost have to go a little backward or you have to let it grow up and you create some real distance with that. (Case-1, 016, Manager)

#### 4.2.2. Contingent benefits and costs of the blended form

The initial strategic priority for Case 3 was to raise the funds and secure the operating relationships (e.g., with suppliers, landlords) to open the first store. Over time, the aim was to gradually transition from early reliance on philanthropic support to sufficient sales revenue to sustain operations. These different sources of material support were associated with distinct stakeholders: (multi)national corporations and their associated foundations provided much of the startup funding; suppliers of discounted or donated stock included local and national companies and nonprofits; the desired consumer base was local residents. The Blended form experienced a mixture of legitimacy bonuses and discounts among these various stakeholders, influenced by national trends affecting the social expectations for business, the local organizational landscape described above, and features of the local sociohistorical context shaping residents' perceptions and expectations.

Regarding access to *material* resources to support the organization during its startup phase, the innovative potential of the Blended form—simultaneously a business and a nonprofit—was central to its appeal to critical resource-providing stakeholders. The promise of self-sufficiency and a retail-based solution to solving the lack of access to healthy food increased its appeal among businesspeople and corporate funders; they, in turn, helped raise its profile, contributed funding, or supplied discounted stock or food. Blended organization senior leaders leveraged their business reputations and networks to enhance this appeal to the business community to support their social mission.

Its mission and proposition is so compelling, and that's the reason [we/company] care so deeply about it and want to make the model work […] you have to run a great business, but I believe you can do that, and affordable nutrition is something we can't give up on. (Case-3, 012, Board Member)

[Founder] has got more influence and connections than I'll ever have. (Case-3, 008, Nonprofit collaborator)

At the same time, being able to signal credibly that it was a nonprofit also helped to boost legitimacy with key nonprofit operating partners, including a local nonprofit landlord and a sizeable food bank that contributed to its supply chain.

I mean we probably gave them a break on rent because of their nonprofit status. (Case-3, 007, Nonprofit collaborator)

Local residents were the would-be consumers who represented both the constituency that the Blended form aimed to serve through healthy, affordable grocery offerings, and on whom it depended for retail revenue to break-even. With this group, legitimacy was more problematic, as the organization's hybrid form conformed to expectations for neither a regular grocery store nor a nonprofit foodbank. As the organization was reliant on donated and reduced cost goods, and limited to stock that met its strict nutritional standards, some products local shoppers expected from a grocery store were not stocked, while others were inconsistently available and priced. This led to shopper critiques and doubts about the organization's legitimacy as a business akin to other grocers.

I'd say the most difficult part of working here is not everybody understands the purpose of the store, so sometimes I feel like a broken record trying to explain things […] why you don't have soda on the shelves or why there's not these certain chips (Case-3, Focus group, Staff)

Some staff believed marketing the organization as a nonprofit would help the local community understand that the low prices being offered were not a scam. However, other staff pointed to doubts about its credentials as a nonprofit, not least because it sold rather than gave away its (partially donated) stock.

Customers will say “I hear that 90% of your stuff is donated. Why are you selling at this price?” (Case 3, 005, Staff).

Moreover, in a city marked by stark inequities and structural racism, the Blended organization had to battle deep local skepticism about both business and nonprofits: skepticism that it was a business looking to profit off the indignities of local food deserts, and skepticism that it was one more nonprofit led by affluent outsiders that would ultimately let down local residents. For example, initial store openings had been delayed because of concerns that the organizational model amounted to selling rotten food to poor people.

It's more this stigma of what they believe that we were about. They believe that we were the “sell you out-of-date [food]” people. (Case-3, Focus group, Staff)

We had a [neighborhood leader] saying “Well geez. This is White people bringing food into this [neighborhood], and if it's so great, why don’t they do it in [founder's neighborhood]”. (Case-3, 006, Board member)

Challenges blending nonprofit and business cultures and balancing revenue-generation and public health goals led to internal debates, which slowed decision-making and which staff found hard to resolve without risking commitment to one or other set of goals. For example, organization members consistently struggled to decide on stock and pricing that struck the right balance between generating sufficient revenue and satisfying the mission.

So from the top down, or from procurement or CEO, they're like, “Yes, we’ll take that donation.” And I'm like, “No, we can’t.” [due to nutritional guidelines] So you know, those things can be difficult. (Case-3, 016, Staff)

It is the nature of our mission that has complicated our life and made it much more difficult (a) to do business (b) to attract customers and (c) to communicate who we are to the community. (Case-3, 018, Manager)

At the time of data collection, retail sales had not grown as rapidly as hoped, requiring more reliance on philanthropic funding than anticipated. This reality was raising questions about whether to continue to pursue aspirations for breaking even through blending business and nonprofit elements or pivot to a more traditional nonprofit form and long-term reliance on philanthropic funding. At the same time, some were also questioning how much longer the organization would be able to secure philanthropic funding in its current form.

I mean that really speaks to like the unique identity crisis that [Case-3] faces, is that we could choose one or the other. We could choose to go full-on nonprofit, and choose to be entirely funded through philanthropy, and just exist. Or we can just choose to be a grocery store, and get rid of our nutrition guidelines that are a huge restriction that we imposed on ourselves, and be profitable that way. (Case-3, 015, Staff)

## 5. Discussion

To harness the potential value of business–nonprofit hybrid organizing to support social purpose initiatives and advance public health, managers and leaders require a greater understanding of the different collaborative forms such hybrid organizing may take, and of the relative merits of different forms. Having distinguished Appended and Blended forms of business–nonprofit hybrids, our findings indicate the necessity of a dynamic model of hybrid organizing, both to optimize the benefits for initiatives targeting social and economic drivers of health and to enable resilience over time.

### 5.1. Comparing appended and blended forms of business–nonprofit hybrid organizing

The Appended form had several advantages as an innovative means of fostering private sector contributions to promoting equitable public health. The differentiated structure, combining integration with a degree of compartmentalization, helped secure resources and legitimacy for the initiative by enabling the responsible unit to conform to key nonprofit norms (4, 17). At the same time, the larger size and resources of the host business enabled their nonprofit elements to break into and establish their initiatives in a competitive nonprofit landscape, by drawing on the established business' networks, reputation, in-kind, and financial resources. These advantages run counter to literature, which emphasizes the costs of asymmetry for nonprofits and achieving social missions (21). As such, our findings challenge overly simplistic views of asymmetrical integration, which focus only on the risks to nonprofits of engaging with resource-rich(er) businesses (14).

Nonetheless*,* we also saw that Appended hybrid organizing became disadvantageous when it threatened the initiative's ability to compete with other initiatives on nonprofit terms and when business–nonprofit asymmetries prevented capitalizing on the complementary knowledge and expertise of business members. These costs became especially problematic as strategic priorities shifted from establishing to scaling up the initiative. This suggests that existing, well-resourced businesses may have an especially valuable role to play in launching and nurturing social purpose initiatives via Appended hybrid organizing.

In contrast to the Appended forms' reliance on conforming to sectoral norms, the Blended form sought to sustain itself by diverging from purely nonprofit or business norms. This innovative mix of being entirely mission-driven *and* financially self-sustaining was critical to its ability to secure the relationships and resources (especially from the corporate sector) to get established. The future social and economic value of this integrated form also helped to sustain the commitment of the CEO and other members to persevere with inherent managerial challenges (17). However, there were two major drawbacks to achieving its mission, both of which were managerial. The first was the complexity of blending business and nonprofit elements into a unified and consistent framework. The second was effectively selling this atypical identity to the local residents it sought to serve and on whom it relied to generate retail revenue. These findings suggest that harnessing the Blended form to achieve the desired social and health impacts may require especially high levels of managerial dexterity. This aligns with existing literature documenting the internal managerial challenges of seamlessly integrating nonprofit and business elements (12, 18) and the relative advantages of compartmentalization to do so (4, 17).

Our findings further suggest, however, that it is not only the capacity to manage tensions internally that managers must consider but also the characteristics of the external stakeholders among whom they seek recognition as legitimate. Specifically, managers must consider how fragmented and legible those stakeholders may be. Key stakeholders for the Appended form included public sector organizations (public schools) and philanthropic funders from the nonprofit or business worlds. Squarely embedded within one or other extant sector (nonprofit, public, or business), where the normative criteria for evaluations of the organization are relatively clear (19), the expectations of these stakeholders were quite clear to managers of the Appended form. Senior leaders of the Blended form also had some success winning legitimacy in the eyes of stakeholders rooted in defined sectors with which they were familiar (e.g., the corporate sector). However, its would-be customers comprised a heterogenous and much less well-defined constituency, which appeared less legible to the Blended organization's managers. In evaluating the relative merits of a given form of hybrid organizing, managers must therefore also invest in efforts to understand and take account of characteristics of the stakeholders on whom their success depends, particularly their anticipated receptivity to an unconventional organizational form.

### 5.2. A dynamic model of optimizing business–nonprofit hybrid organizing

While each hybrid collaborative form has certain characteristics and related benefits and costs, recognition of the context-specific nature of these benefits and costs is central to our contribution. For each form, the significance of benefits and costs depended on interrelated and dynamic factors, including changes in resource needs and the relative importance of different external stakeholders as strategic priorities evolved, and features of the context that could heighten or attenuate these benefits and costs. This dynamic model of the balance of trade-offs (Figure 1) makes clear that optimizing hybrid organizing for public health entails an ongoing process of assessing and seeking fit between form and context in ways that allow the form to evolve. Further, this implies a need to move away from implicitly normative frameworks wherein the trajectory of evolution is always oriented toward greater integration, while separation represents failure or abandonment (22, 31). Rather, the optimal form may differ for different types of organizations and at different times. Thus, determining the optimal form of hybrid organizing is not a one-time decision, but a decision that requires continual review. Moreover, scholars and practitioners must recognize that separation may represent a positive evolution and can entail productive, ongoing relationships in a different collaborative form. Further research is needed to better understand how to optimize evolution of the collaborative form at later stages than our cases allowed (32), including research that elucidates pathways to successful separation.

This dynamic model also advances an understanding of the double-edged nature of hybrid organizing in relation to legitimacy bonuses and discounts, specifically, the importance of attending to the *temporal* dimension of legitimacy dynamics and the ways in which changes in strategic priorities over time (e.g., launching vs. scaling an initiative) influence how consequential the perceptions of different stakeholders are to achieving the organizations' mission. For example, as the Blended form sought to increase the proportion of revenue generated through sales, local residents' views of the legitimacy of this collaborative form became more significant, prompting a consideration of the need to evolve the approach to hybrid organizing.

In addition to strategic priorities, our model identifies contextual features as another set of factors for managers to consider in navigating the ongoing assessment of fit. Previous literature has noted how wider societal trends may be driving a greater acceptability of organizational forms that span sectoral boundaries (6), perhaps even institutionalizing hybrid forms as a distinct fourth sector (33). Our study highlights the significance of *local* organizational and socioeconomic influences on hybrid forms. Building on Marquis and colleague's (34) work, our findings suggest that community isomorphism, i.e., the resemblance of a corporation's social practices to those of other corporations within its community, is important in shaping the perceived merits of different forms of business–nonprofit hybrid organizing. In this case, community isomorphism appeared to galvanize business’ willingness to support hybrid organizing, while features of the social and historical context further complicated the Blended form's difficulties, successfully appealing to the local residents. Hence when assessing fit with context, practitioners need to attend not only to national societal or business trends but also to specific local social histories and organizational landscapes, and how they may intersect.

Of course, contexts are themselves dynamic. Although outside our study frame, the current moment—including the impacts of a global pandemic and intensifying movement for racial justice—has created exogenous shocks that could precipitate changes in the balance of trade-offs. For example, such shocks might affect the availability of funding from particular sources, galvanize business' interest in contributing to public health, or intensify skepticism and distrust of certain institutions. Understanding the impacts of these contemporary changes in the operating environment will require further investigation. Nonetheless, in contrast to static models of the hybrid organization, our dynamic model provides a valuable foundation for enabling resilience through an evolution of hybrid organizing by orienting to a continual assessment of fit between context, strategic priorities, and merits of a given form.

### 5.3. Study limitations

Our study was limited to three cases in a single context. The study included more data about the Appended form than the Blended, reflecting differences in the size of organizations, and identification of two Appended cases (because identification of the form was an output of analysis and not part of the selection criteria). The forms we identified may not be exhaustive. Other forms of business–nonprofit hybrid organizing, and similar forms in other contexts, may expand the range of the strengths and weaknesses identified and/or highlight additional factors on which the balance of trade-offs is contingent. We purposefully selected initiatives that were successfully sustained; an examination of less successful collaborations may generate additional insights into associated risks. Cases with even longer trajectories could further illuminate evolution and transitions between forms of hybrid organizing. Further, in focusing on comparison and distinctions between cases, we attended less to what made each trajectory unique (e.g., differences between Appended cases).

Additionally, measuring the impact of initiatives and how impact related to forms of organizing was beyond our scope. Moreover, a consideration of social value may be important for how practitioners weigh the relative benefits and costs of different forms. For example, the challenges associated with the Blended form may be considered more tolerable because of the form's transformative potential to tackle underlying structural drivers, e.g., addressing market failures that result in inequitable access to affordable healthy food.

## 6. Conclusion

Despite the proliferation of hybrid organizing at the interface of nonprofit and business sectors, existing typologies of cross-sector collaboration inadequately differentiate the diverse forms that deep business–nonprofit integration may take. Moreover, the relative merits of these forms, the contradictory mix of associated legitimacy discount and bonuses, and the conditions under which costs or benefits may be more or less salient are not well understood (4, 5). In this paper, we have contributed to the conceptual development of existing typologies of collaboration by describing two distinct integrated forms of business–nonprofit hybrid organizing—Appended and Blended—and by characterizing the potential benefits and costs associated with each form. The differences we highlighted in the pattern of relative costs and benefits underscore the importance of differentiating between forms of business–nonprofit hybrid organizing. Our findings also point to different ways in which businesses can contribute to collaborations targeting complex public health issues, via different roles in establishing, nurturing, sustaining, and/or spinning off social purpose initiatives into independent ventures.

Equally important, however, is that our findings demonstrate that the costs and benefits of these different collaborative forms are not static or fixed traits. Rather, we have outlined a dynamic model (depicted in Figure 1) in which the optimal form of hybrid organizing depends on the interplay between the characteristics of the form, the strategic priorities of the initiative, and the relevant features of the operating environment. This model has several implications for organizational theory and for managers interested in harnessing the potential of deeper business–nonprofit integration to impact public health. First, it makes clear the importance of avoiding normative typologies which suggest that more vs. less integration, or any particular form of hybrid organizing, is inherently better. Instead, it draws attention to the contingent nature of benefits and costs, and to the potential for positive evolution to be toward more *or* less an integration of nonprofit and business elements over time. Second, this model adds a more nuanced understanding to the contradictory picture of coexisting benefits and costs by helping to identify some of the conditions under which the benefits or costs of hybrid organizing become more or less valuable or problematic for establishing and sustaining a social purpose initiative. Third, as a framework for decision-making, it directs the attention of practitioners to the importance of an ongoing process of assessing and seeking fit and outlines a set of factors that managers should consider as they weigh the balance of the trade-offs of different forms of business–nonprofit hybrid organizing. Fourth, in foregrounding a dynamic view of business–nonprofit hybrid organizing, this model promotes an orientation to evolving and adapting collaborative forms in order to ensure resilience and the ability to sustain efforts to promote health equity for the long term (35).

## Data Availability

Anonymized data that support the findings of this study are available on reasonable request from the authors. The dataset generated and analyzed during the current study are not publicly available since they contain information that could compromise research participant privacy/consent.

## Footnotes

^1^Aveling E-L, Roberts J, Taylor L, Edmondson A, Singer S. Racial justice and health equity demand a “whole company” approach. *Forbes (Under Review)*. (2020).
